# Effects of Combined Calcium and Vitamin D Supplementation on Insulin Secretion, Insulin Sensitivity and β-Cell Function in Multi-Ethnic Vitamin D-Deficient Adults at Risk for Type 2 Diabetes: A Pilot Randomized, Placebo-Controlled Trial

**DOI:** 10.1371/journal.pone.0109607

**Published:** 2014-10-09

**Authors:** Claudia Gagnon, Robin M. Daly, André Carpentier, Zhong X. Lu, Catherine Shore-Lorenti, Ken Sikaris, Sonia Jean, Peter R. Ebeling

**Affiliations:** 1 Department of Medicine, NorthWest Academic Centre, The University of Melbourne, Western Health, Melbourne, Australia; 2 Department of Medicine, University of Sherbrooke, Sherbrooke, Canada; 3 Melbourne Pathology, Melbourne, Australia; 4 Department of Medicine, Monash University, Melbourne, Australia; 5 Department of Chronic Diseases Surveillance, Institut national de santé publique du Québec, Quebec City, Canada; 6 Department of Medicine, Laval University, Quebec City, Canada; Bielefeld Evangelical Hospital, Germany

## Abstract

**Objectives:**

To examine whether combined vitamin D and calcium supplementation improves insulin sensitivity, insulin secretion, β-cell function, inflammation and metabolic markers.

**Design:**

6-month randomized, placebo-controlled trial.

**Participants:**

Ninety-five adults with serum 25-hydroxyvitamin D [25(OH)D] ≤55 nmol/L at risk of type 2 diabetes (with prediabetes or an AUSDRISK score ≥15) were randomized. Analyses included participants who completed the baseline and final visits (treatment *n* = 35; placebo *n* = 45).

**Intervention:**

Daily calcium carbonate (1,200 mg) and cholecalciferol [2,000–6,000 IU to target 25(OH)D >75 nmol/L] or matching placebos for 6 months.

**Measurements:**

Insulin sensitivity (HOMA2%S, Matsuda index), insulin secretion (insulinogenic index, area under the curve (AUC) for C-peptide) and β-cell function (Matsuda index x AUC for C-peptide) derived from a 75 g 2-h OGTT; anthropometry; blood pressure; lipid profile; hs-CRP; TNF-α; IL-6; adiponectin; total and undercarboxylated osteocalcin.

**Results:**

Participants were middle-aged adults (mean age 54 years; 69% Europid) at risk of type 2 diabetes (48% with prediabetes). Compliance was >80% for calcium and vitamin D. Mean serum 25(OH)D concentration increased from 48 to 95 nmol/L in the treatment group (91% achieved >75 nmol/L), but remained unchanged in controls. There were no significant changes in insulin sensitivity, insulin secretion and β-cell function, or in inflammatory and metabolic markers between or within the groups, before or after adjustment for potential confounders including waist circumference and season of recruitment. In a post hoc analysis restricted to participants with prediabetes, a significant beneficial effect of vitamin D and calcium supplementation on insulin sensitivity (HOMA%S and Matsuda) was observed.

**Conclusions:**

Daily vitamin D and calcium supplementation for 6 months may not change OGTT-derived measures of insulin sensitivity, insulin secretion and β-cell function in multi-ethnic adults with low vitamin D status at risk of type 2 diabetes. However, in participants with prediabetes, supplementation with vitamin D and calcium may improve insulin sensitivity.

**Trial Registration:**

Australian New Zealand Clinical Trials Registry ACTRN12609000043235

## Introduction

Low serum 25-hydroxyvitamin D [25(OH)D] concentrations and dietary calcium intake have been associated with impaired insulin sensitivity or secretion in people at high risk of type 2 diabetes [1], [2]. Furthermore, several prospective epidemiological studies [3], including our previous research [4], have shown that low serum 25(OH)D concentrations are associated with an increased risk of type 2 diabetes. However, data from randomized controlled trials (RCTs) of vitamin D and/or calcium supplementation have been inconclusive. Some studies have reported a beneficial effect on insulin sensitivity [5]–[9] and/or secretion [6], [10], [11] whereas several others failed to demonstrate any effect [12]–[23]. Among these studies, only one trial over 16 weeks has investigated the combined effects of calcium and vitamin D supplementation on insulin sensitivity and secretion in people at high risk of type 2 diabetes [10]. It is thus clear that further well-designed longer-term clinical trials are needed to evaluate the efficacy of combined calcium and vitamin D supplementation on type 2 diabetes risk.

The primary aim of this 6-month, double-blinded, placebo-controlled randomized trial was to evaluate whether combined calcium and vitamin D supplementation could improve insulin sensitivity, insulin secretion and β-cell function in vitamin D-deficient individuals at risk of type 2 diabetes. Secondary aims were to evaluate the effects of supplementation on anthropometry, blood pressure, the lipid profile, inflammation and metabolic markers.

## Materials and Methods

### Study design

This was a 6-month, double-blinded, placebo-controlled trial conducted at a single site (Western Hospital, Melbourne, Australia) in which 95 participants stratified by age (<50 or ≥50 years), sex and BMI (<30 or ≥30 kg/m^2^) in blocks of ten (block-stratified randomization) were randomly allocated in a 1∶1 ratio to either combined calcium and vitamin D treatment or placebo. Randomization was conducted by an independent researcher using computer-generation randomization of study numbers (Microsoft Excel). All investigators, research staff and participants were blinded to the treatment allocation. The protocol for this trial and supporting CONSORT checklist are available as supporting information; see Checklist S1 and Protocol S1.

### Participants

Multi-ethnic vitamin D-deficient men and women aged ≥18 years and at risk of type 2 diabetes were recruited through newspaper and radio advertisements, flyers posted in hospitals and medical clinics as well as presentations given to local community groups in the western Melbourne metropolitan area (Australia) between January 2009 and February 2011. Participants were included if they had a BMI between 25 and 40 kg/m^2^, a serum 25(OH)D concentration ≤50 nmol/L [24] and a diagnosis of prediabetes (fasting plasma glucose 6.1–6.9 mmol/L and/or 2-h plasma glucose post 75 g glucose load 7.8–11.0 mmol/L). During the trial, changes in the inclusion criteria were approved by the ethics committee to improve the recruitment rate: participants were included if they had a serum 25(OH)D concentration ≤55 nmol/L and if they either had a diagnosis of prediabetes or scored ≥15 on the Australian Type 2 Diabetes Risk Assessment tool (AUSDRISK). The AUSDRISK is a validated and simple 10-point questionnaire that predicts the risk of type 2 diabetes (http://www.health.gov.au/internet/main/publishing.nsf/Content/chronic-diab-prev-aus). A score of 15 corresponds to a 10% probability of diabetes in the next 5 years.

Participants were excluded based on the following criteria: HbA_1c_ ≥6.5%, pregnancy or breast-feeding, creatinine clearance <60 ml/min, cirrhosis, malabsorption or elevated serum anti-tissue transglutaminase antibody, hypercalcemia, hypercalciuria, history of nephrolithiasis, previous non traumatic fractures, serum 25(OH)D <13 nmol/L, active or chronic inflammation (based on clinical assessment), medications known to affect glucose and mineral metabolism over the last 3 months, pharmacological treatment for obesity, commencement of physical activity ≥3 times/week or >5% change in weight in the last 3 months.

The study was approved by the Melbourne Health Human Research Ethics Committee and the procedures followed were in accordance with the ethical standards of the institution and the declaration of Helsinki. The study was registered with the Australian New Zealand Clinical Trials Registry (ACTRN12609000043235: https://www.anzctr.org.au/Trial/Registration/TrialReview.aspx?ACTRN=12609000043235). All participants provided written informed consent.

### Intervention

The treatment group commenced on 2,000 IU of vitamin D_3_ (two capsules of 1,000 IU of OstevitD, Key Pharmaceuticals, Australia) plus 1,200 mg of elemental calcium (two capsules of calcium carbonate, Wyeth Consumer Healthcare, Australia) daily with breakfast. After 2 and 4 months, serum 25(OH)D was measured and if necessary, the vitamin D_3_ dose was increased by 2,000 IU every 2 months to reach a target of ≥75 nmol/L. Serum 25(OH)D results were sent directly to the Western Health clinical trial pharmacist who was responsible for applying the protocol. The pharmacist dispensed all study supplements and performed capsule count every 2 months. To maintain blinding, extra vitamin D placebo capsules were provided to participants to match a total of four and six capsules starting at 2 and 4 months, respectively. The placebo group was given matching calcium placebo capsules as well as 2, 4 and 6 vitamin D placebo capsules daily at 0, 2 and 4 months, respectively (Stenlake Compounding Chemist, Australia). Every 2 months, participants were asked about a change in their medical condition or about side effects including symptoms of vitamin D hypersensitivity. Finally, a colleague not involved in the study performed safety monitoring of serum calcium every 2 months.

### Measurements

The following measurements were performed at baseline and 6 months, unless stated.

### Anthropometry and blood pressure

Weight, height, waist circumference and blood pressure were assessed every 2 months in the morning before performing the biochemical tests. Weight was measured to the nearest 0.1 kg, without shoes and in light clothing, using an electronic calibrated scale. Height was measured to the nearest 0.1 cm using a wall-mounted stadiometer. BMI was calculated in kg/m^2^. Waist circumference was measured in duplicate to the nearest 0.5 cm using a tape placed at the midpoint between the lower rib and the upper iliac crest, at the end of a normal expiration. Seated blood pressure was measured in triplicate, after 5 minutes of rest in a quiet room. For all measures, the average of multiple measurements was calculated.

### Diet, physical activity and sunlight exposure

To estimate dietary calcium and vitamin D intake, a calcium-specific food frequency questionnaire, developed and validated in Australia, was adapted by adding major dietary sources of vitamin D [25]. Daily intakes were estimated using Australian-specific dietary software (FoodWorks, Xyris Software, Australia). The short International Physical Activity Questionnaire was used to estimate physical activity habits over the last week. The sum of metabolic equivalent of task (MET)-minutes per week spent walking and doing moderate and vigorous physical activity was calculated based on the updated Compendium of Physical Activities [26] and the 2005 IPAQ guidelines (http://www.ipaq.ki.se/scoring.pdf). A sun exposure questionnaire was administered to estimate weekly exposure to sunlight over the last 2 months. Participants were asked how many hours per week they spent in the sun between 9 am and 5 pm, how often they used sunscreen (≥50% or <50% of the time) and the degree of clothing worn when outside (shirt/hat/pants/skirt) to determine the fraction of body surface area exposed to sunlight. A sun index was calculated as follows: hours of sun exposure/week multiplied by the fraction of the body surface area exposed. Based on the month of blood testing at baseline, participants were divided into two groups: spring/summer (in Australia from October to March) and fall/winter (from September to April).

### Measures of insulin sensitivity, insulin secretion and β-cell function

A 75-g 2-h oral glucose tolerance test (OGTT) was performed in the morning after a 12-h fast. Venous blood was collected through a catheter at −30, −20, −10, 0, 10, 20, 30, 60, 90 and 120 minutes for the determination of plasma glucose, serum insulin and C-peptide. Measures at times −30, −20, −10 and 0 minutes were averaged to derive fasting values. HOMA2%S and Matsuda indices were used to evaluate insulin sensitivity. HOMA2%S was calculated using the HOMA calculator (http://www.dtu.ox.ac.uk) while Matsuda was calculated with the following formula: 10,000 divided by the square root of [(fasting glucose x fasting insulin) x (mean glucose^30–120 min^ x mean insulin^30–120 min^)]. The insulinogenic index (delta C-peptide^0–30 min^ divided by delta glucose^0–30 min^) and the area under the curve (AUC) for C-peptide were used to estimate insulin secretion. β-cell function was assessed using the disposition index and calculated by multiplying Matsuda index by AUC for C-peptide. Glucose was measured enzymatically by the hexokinase method (Roche c701), while insulin and C-peptide were measured by electrochemiluminescence immunoassays (Roche e602).

### Serum 25-hydroxyvitamin D, parathyroid hormone, inflammatory and metabolic markers

Serum was stored at −80°C until measurements were performed in a single batch for each measure at the end of the study. All biochemical markers were assessed at 0 and 6 months, except for serum 25(OH)D which was assessed every 2 months. Serum 25(OH)D was analysed by an automated chemiluminescent immunoassay (DiaSorin, USA, CV 6.6%). Parathyroid hormone was assessed by electrochemiluminescence immunoassay (Roche e602) and high-sensitive C-reactive protein (hs-CRP) by immunoturbidimetric immunoassay (Roche c701). Lipid profiles were measured using standard methods (Roche c701). Serum IL-6 and TNF-α were measured by a chemiluminescent enzyme immunoassay (Immulite, Siemens, USA). Glycated haemoglobin (HbA_1c_) was assessed by a DCCT-aligned ion exchange HPLC method (Bio-Rad Variant II Turbo 2.0 analysers, CV 1.6%). Adiponectin was measured by ELISA (R&D Systems, USA) and total osteocalcin by the automated Elecsys assay (Roche, Australia). Undercarboxylated osteocalcin was measured using the same automated assay described for total osteocalcin. However, measurement was preceded by sample pre-treatment with a 100 mg/mL hydroxyapatite slurry, following the method described by Gundberg [27].

### Statistical analysis

Analyses were performed using SAS statistical software version 9.2 (SAS Institute, Cary, NC, USA). Data were analyzed on all participants who completed the baseline and final visits regardless of compliance. No data was imputed for the few participants with missing data (*n* = 4). Data not following normal distribution were log-transformed prior to analysis. Baseline characteristics of the participants were compared between treatment groups using an unpaired *t* test or a chi-square test, as appropriate. A multivariate 2-factor repeated-measures regression analysis was used to assess time effects and time-by-treatment interactions effect on all outcome measures after adjustment for the variables that were different between treatment groups at baseline with a *P* value ≤0.10 (fasting glucose, waist circumference, season of recruitment, prediabetes status, systolic and diastolic blood pressure, hs-CRP, IL-6, current smoking and dietary vitamin D intake). For IL-6, which was non-normally distributed after transformation, an ANOVA on rank was used to detect between-group differences after adjusting for variables that were different between groups at baseline. A post hoc analysis was performed on the participants who had prediabetes based on the first study visit 2-h OGTT. This analysis thus excluded participants with normal glucose tolerance or newly diagnosed type 2 diabetes. In this subgroup, baseline characteristics of the participants were not different between treatment groups and thus unadjusted data are presented.

Based on the difference in delta HOMA-IR [0.15±0.18 (SD)] observed in a population of overweight men and women with prediabetes before and after a mean weight loss of 9.8 kg [28], we initially calculated that we would require a total of 80 participants to detect this difference in HOMA-IR (that was considered clinically significant) with a power of 90%, a 2-sided alpha of 0.05 and a dropout rate of 20%. Sample size estimates were then recalculated based on a secondary analysis of a 3-year RCT which reported that combined calcium and vitamin D supplementation was associated with a 0.86 (standard deviation of 1.7) difference for the change in HOMA-IR relative to placebo in older adults with impaired fasting glucose [29]. Based on these findings, we estimated that we would require 160 participants (80 per group) to detect a difference of this magnitude at 80% power with a 2-sided alpha of 0.05, and assuming a dropout rate of 20%. However, due to slower participant recruitment than anticipated, recruitment was stopped after the 95^th^ participant was randomized.

## Results

### Participant characteristics

**Figure 1:**
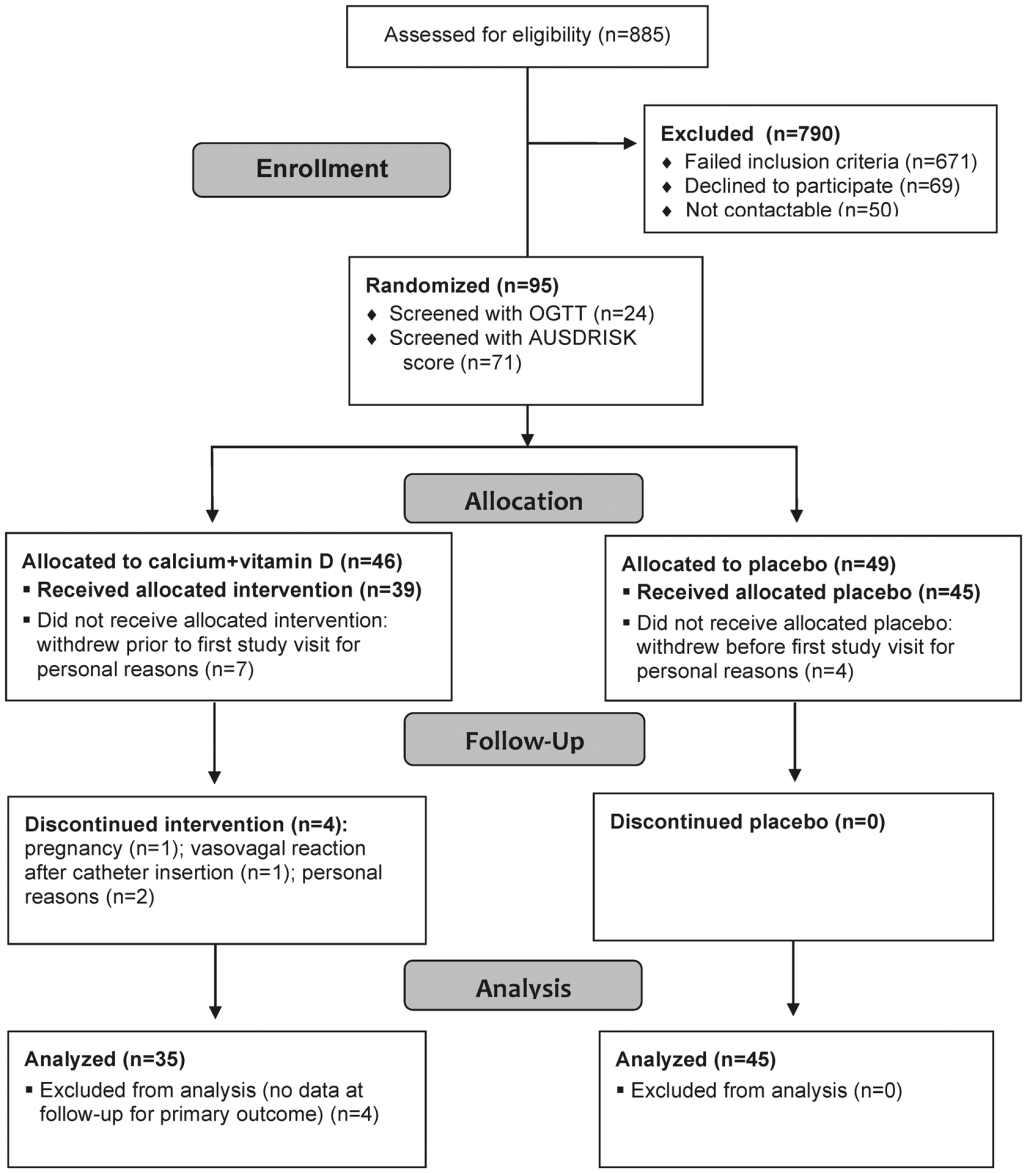
Flowchart showing participant enrollment, allocation, follow-up and analysis.

A flowchart showing participant enrollment, allocation, follow-up and analysis is presented in **Figure 1**. A total of 885 people were telephone-screened, of whom 510 were invited to undergo further screening. Ninety-five participants met the study criteria and were randomized (24 were screened with OGTT and 71 with AUSDRISK questionnaire). For administrative purposes, randomization was performed 24 to 72 h before the first study visit. Eleven participants withdrew from the study for personal reasons (not interested anymore) between randomization and the first study visit (treatment *n* = 7; placebo *n* = 4), and thus had no baseline data, leaving 84 participants who completed the baseline study visit (treatment *n* = 39; placebo *n* = 45). Moreover, four participants did not complete the last study visit (treatment *n* = 4). Reasons for withdrawal during the study were: pregnancy (*n* = 1), vasovagal reaction after attempts to insert a catheter during the last study visit (*n* = 1) and personal reasons (*n* = 2). Comparison of the 15 participants who withdrew and the 80 participants who completed the study revealed that those who withdrew were significantly younger (mean age 46 vs. 55 years, *P* = 0.03), but there was no difference between the groups for sex, ethnicity, serum 25(OH)D concentrations, BMI, waist circumference and AUSDRISK score at baseline.

Baseline characteristics of the 80 participants who completed the baseline and final study visits are presented in **Table 1**. Participants were on average 54 years of age, had a mean BMI of 31 kg/m^2^ and were mostly women of European background. After inclusion, an OGTT was performed. Diabetes was diagnosed in 14% of the participants (*n* = 11), 39% were glucose tolerant (*n* = 31) and 48% had prediabetes (impaired fasting glucose and/or impaired glucose tolerance) (*n* = 38). Baseline characteristics were similar between treatment groups, with the exception that those in the treatment group were more likely to be current smokers, to start the study during spring/summer, to have higher dietary vitamin D intake and have lower waist circumference, systolic and diastolic blood pressure, fasting plasma glucose, hs-CRP and IL-6 concentrations. They were also less likely to have prediabetes.

**Table 1:** Baseline characteristics of the participants by treatment group.

Characteristic	Treatment (*n* = 35)	Placebo (*n* = 45)	*P* value*
Age, years	53.8 (11.9)	55.3 (11.1)	0.57
Women, %	71	67	0.65
Ethnicity, %			0.39
*European background*	69	69	
*Asian*	23	29	
*Other*	9	2	
Season of recruitment, %			0.003
*Spring/summer (Oct-Mar)*	94	67	
*Fall/winter (Apr-Sep)*	6	33	
Current smoking, %	17	2	0.02
First degree relative with diabetes, %	66	76	0.23
AUSDRISK score†	18.5 (3.3)	18.9 (3.3)	0.57
BMI†, kg/m^2^	31.1 (5.7)	31.9 (6.2)	0.61
Waist circumference, cm	103.0 (11.5)	108.4 (15.3)	0.08
Systolic blood pressure, mm Hg	120.8 (14.0)	126.8 (12.1)	0.05
Diastolic blood pressure, mm Hg	73.1 (10.0)	76.8 (8.9)	0.09
HbA_1c_, %	5.6 (0.4)	5.7 (0.4)	0.31
Fasting glucose, mmol/L	5.2 (0.5)	5.5 (0.6)	0.04
Fasting insulin, µU/mL	12.6 (7.1)	15.2 (7.6)	0.11
Prediabetes‡, %	37	56	0.10
*Impaired fasting glucose (≥6.1 mmol/L), %*	0	11	0.04
*Impaired glucose tolerance, %*	37	53	0.15
25-hydroxyvitamin D, nmol/L	47 (13)	43 (13)	0.25
Parathyroid hormone†, pmol/L	5.3 (2.0)	5.5 (1.8)	0.63
Corrected calcium, mmol/L	2.15 (0.08)	2.16 (0.08)	0.59
Serum creatinine, µmol/L	68 (12)	69 (13)	0.90
Dietary vitamin D intake†, IU/day	132 (76)	108 (60)	0.08
Dietary calcium intake, mg/day	689 (419)	563 (275)	0.13
Physical activity, MET-min/week	2171 (3238)	1811 (2428)	0.55
Time spent outdoor†, h/day	1.8 (1.4)	1.8 (1.5)	0.88
Sun index†, h/week/m^2^	158 (233)	152 (218)	0.58
Sunscreen use <50% of the time, %	66	69	0.76

Data are presented as mean (SD) or %.

*Unpaired *t* test or chi-square test, as appropriate.

† Logarithmically-transformed variables.

‡ Comprises participants with impaired fasting glucose and/or impaired glucose tolerance.

BMI, body mass index; MET, metabolic equivalent of task.

### Supplement compliance and change in serum 25(OH)D concentration

**Figure 2:**
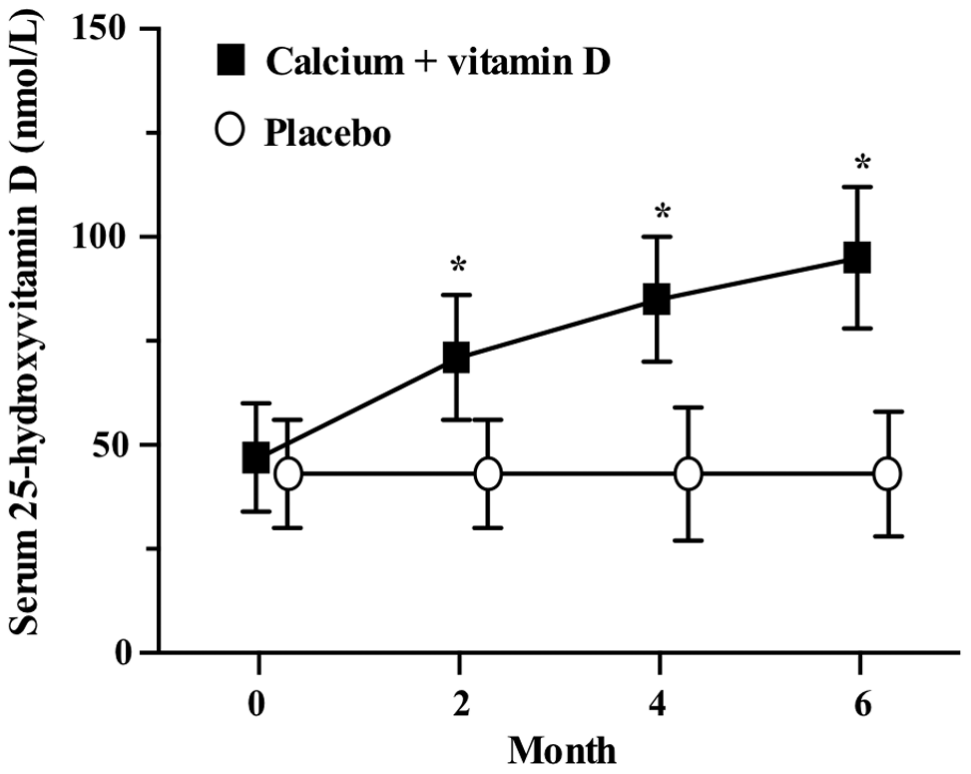
Mean (SD) serum 25-hydroxyvitamin D concentrations at baseline and after 2, 4 and 6 months in the calcium and vitamin D treatment and placebo group. *P<0.0001 for the difference between groups.

Supplement compliance was similar between groups for calcium (treatment 81%; placebo 76%) and vitamin D (treatment 85%; placebo 80%). After 6 months, serum 25(OH)D increased by a mean of 48 nmol/L (95% CI, 41–56 nmol/L) to reach a mean concentration of 95 nmol/L (95% CI, 89–101 nmol/L) in the treatment group; there was no change in the control group (**Figure 2**). Overall, 91% of the participants in the treatment group reached the target serum 25(OH)D of 75 nmol/L. Most participants required 4,000 IU/d of vitamin D to reach the target (*n* = 22) while a smaller proportion needed 2,000 IU/d (*n* = 9) or 6,000 IU/d (*n* = 4).

### Changes in insulin sensitivity, insulin secretion, β-cell function, metabolic and inflammatory markers

There were no differences in the changes in any of the insulin sensitivity or secretion measures, or in the disposition index, between the treatment and placebo groups, even after taking into account the variables that were different between groups at baseline (fasting glucose, waist circumference, season of recruitment, prediabetes status, systolic and diastolic blood pressure, hs-CRP, IL-6, current smoking and dietary vitamin D intake) (**Table 2**). Similarly, waist circumference and blood pressure remained stable for both groups during the intervention. There was however a borderline significant time-by-treatment interaction for body weight due to a small but non-significant 0.49 kg increase in the placebo group that was observed only in the adjusted model (**Table 3**). There were no between group differences in blood lipids, markers of inflammation, adiponectin, total and undercarboxylated osteocalcin concentrations (**Table 4**). In a post hoc analysis restricted to participants with prediabetes, there was a significant between group difference for the change in insulin sensitivity, as assessed by HOMA%S and Matsuda, in favor of the vitamin D and calcium supplementation group (**Table 5**). There were however no between group differences for the change in markers of insulin secretion or β-cell function.

**Table 2:** Mean baseline and 6 month values and the mean absolute changes in insulin sensitivity, insulin secretion and β-cell function in the treatment and placebo group.

			Mean Change	*P* value*		
	Baseline	6 months	(95% CI)	Time	Treatment	Interaction
**HOMA2%S** †						
Treatment (*n* = 34)	63.9 (29.0)	61.1 (25.6)	−2.8 (−9.2, 3.5)	0.01 (0.29)	0.96 (0.10)	0.11 (0.68)
Placebo (*n* = 45)	55.1 (30.4)	52.5 (27.7)	−2.6 (−8.2, 3.0)			
**Matsuda index**						
Treatment (*n* = 33)	49.0 (25.8)	47.3 (21.5)	−1.7 (−7.3, 3.8)	0.40 (0.37)	0.89 (0.12)	0.59 (0.87)
Placebo (*n* = 45)	40.5 (25.8)	39.3 (22.3)	−1.2 (−5.1, 2.8)			
**Insulinogenic index, µU/mL/mmol/L μ**µU/mL µU/mL						
Treatment (*n* = 33)	0.51 (0.28)	0.52 (0.25)	0.01 (−0.07, 0.10)	0.22 (0.67)	0.47 (0.47)	0.67 (0.89)
Placebo (*n* = 45)	0.55 (0.23)	0.56 (0.30)	0.01 (−0.05, 0.06)			
**AUC for C-peptide, nmol/L ×120 min**						
Treatment (*n* = 33)	410.8 (138.8)	418.0 (123.2)	7.2 (−14.1, 28.5)	0.71 (0.97)	0.39 (0.10)	0.75 (0.44)
Placebo (*n* = 43)	463.8 (132.7)	455.8 (97.3)	−8.0 (−38.6, 22.7)			
**Disposition index, nmol/L ×120 min** ‡						
Treatment (*n* = 33)	17 568 (4 936)	17 863 (5 010)	295 (−861, 1 451)	0.90 (0.70)	0.30 (0.09)	0.71 (0.31)
Placebo (*n* = 43)	16 926 (7 605)	16 656 (7 356)	−270 (−1 502, 960)			

Baseline and 6 month data are presented as mean (SD).

*A multivariate 2-factor repeated-measures regression analysis was used to assess time effects and time by treatment effect interactions on all outcome variables after adjustment for the variables that were different between treatment groups at baseline. Numbers in brackets represent unadjusted *P* values.

† Logarithmically-transformed variables.

‡ Calculated by multiplying Matsuda index by AUC for C-peptide.

AUC, area under the curve; HOMA2%S, Homeostasis Model Assessment 2 index of insulin sensitivity.

**Table 3:** Mean baseline values and the mean absolute changes in anthropometry and blood pressure in the treatment and placebo group.

		Mean Change from Baseline (95% CI)			*P* value*		
	Baseline	▵ 2 months	▵ 4 months	▵ 6 months	Time	Treatment	Interaction
**Weight, kg**							
Treatment (*n* = 35)	85.7 (18.2)	−0.05 (−0.56, 0.46)	−0.03 (−0.75, 0.69)	−0.02 (−0.79, 0.75)	0.13 (0.41)	0.68 (0.35)	0.05 (0.29)
Placebo (*n* = 45)	87.9 (21.2)	0.69 (0.31, 1.07)	0.54 (−0.06,1.14)	0.49 (−0.27, 1.25)			
**WC, cm**							
Treatment (*n* = 35)	103.0 (11.5)	0.56 (−0.29, 1.42)	0.30 (−0.51, 1.11)	−0.85 (−1.78, 0.07)	0.66 (<0.01)	0.28 (0.06)	0.69 (0.95)
Placebo (*n* = 45)	108.4 (15.3)†	0.84 (−0.01, 1.69)	0.22 (−0.48, 0.93)	−0.59 (−1.60, 0.42)			
**SBP, mmHg**							
Treatment (*n* = 35)	120.8 (14.0)	−0.11 (−4.90, 4.68)	0.91 (−3.73, 5.56)	2.44 (−0.95, 5.83)	0.06 (0.99)	0.38 (0.11)	0.68 (0.39)
Placebo (*n* = 45)	126.8 (12.1)†	−0.49 (−4.16, 3.19)	−1.20 (−5.10, 2.69)	−2.18 (−6.16, 1.80)			
**DBP, mmHg**							
Treatment (*n* = 35)	73.1 (10.0)	−0.11 (−4.15, 3.92)	−0.66 (−5.65, 4.32)	−0.20 (−3.71, 3.30)	0.38 (0.54)	0.24 (0.23)	0.31 (0.30)
Placebo (*n* = 45)	76.8 (8.9)†	2.24 (−0.67, 5.15)	−1.36 (−3.93, 1.22)	−1.56 (−4.33, 1.22)			

Baseline data are presented as mean (SD).

*A multivariate 2-factor repeated-measures regression analysis was used to assess time effects and time by treatment effect interactions on all outcome variables after adjustment for the variables that were different between treatment groups at baseline. Numbers in brackets represent unadjusted *P* values.

† *P*<0.1 for the difference between groups at baseline (unpaired *t* test or Wilcoxon, as appropriate).

DBP, diastolic blood pressure; SBP, systolic blood pressure; WC, waist circumference.

**Table 4:** Mean baseline and 6 month values and the mean absolute changes in lipid profile, cytokines, adiponectin and osteocalcin in the treatment and placebo group.

			Mean Change	*P* value*		
	Baseline	6 months	(95% CI)	Time	Treatment	Interaction
**Total cholesterol, mmol/L** †						
Treatment (*n* = 32)	5.16 (1.19)	5.10 (1.36)	−0.06 (−0.39, 0.28)	0.80 (0.01)	0.21 (0.45)	0.42 (0.11)
Placebo (*n* = 42)	5.11 (0.99)	4.69 (1.06)	−0.42 (−0.65, −0.19)			
**Triglycerides, mmol/L** †						
Treatment (*n* = 32)	1.56 (0.96)	1.62 (0.93)	0.06 (−0.20, 0.33)	0.47 (0.36)	0.24 (0.92)	0.32 (0.09)
Placebo (*n* = 42)	1.57 (0.72)	1.41 (0.67)	−0.16 (−0.33, 0.01)			
**HDL-cholesterol, mmol/L**						
Treatment (*n* = 32)	1.28 (0.29)	1.27 (0.33)	−0.01 (−0.07, 0.05)	0.91 (0.98)	0.81 (0.75)	0.47 (0.61)
Placebo (*n* = 42)	1.29 (0.33)	1.31 (0.41)	0.02 (−0.06, 0.09)			
**LDL-cholesterol, mmol/L**						
Treatment (*n* = 32)	3.17 (0.99)	3.11 (1.19)	−0.06 (−0.34, 0.23)	0.68 (0.01)	0.26 (0.35)	0.29 (0.36)
Placebo (*n* = 42)	3.10 (0.93)	2.76 (0.91)	−0.34 (−0.53, −0.17)			
**Hs-CRP, mg/L** †						
Treatment (*n* = 34)	5.52 (10.82)	3.62 (5.68)	−1.90 (−5.14, 1.34)	0.97 (0.54)	0.07 (0.10)	0.80 (0.51)
Placebo (*n* = 45)	2.44 (2.15) ‡	2.57 (2.66)	0.13 (−0.31, 0.57)			
**TNF-α, pg/mL** †						
Treatment (*n* = 34)	6.31 (2.18)	6.60 (1.43)	0.29 (−0.28, 0.87)	0.82 (0.02)	0.56 (0.29)	0.39 (0.83)
Placebo (*n* = 45)	6.70 (2.43)	7.10 (2.27)	0.40 (−0.17, 0.97)			
**IL-6, pg/mL** §						
Treatment (*n* = 34)	1.2 (1.2, 2.1)	1.3 (1.2, 1.8)	0.0 (−0.4, 0.1)	NA	0.44 (0.42)	NA
Placebo (*n* = 45)	1.8 (1.4, 2.4) ‡	1.9 (1.4, 2.8)	0.0 (−0.4, 0.5)			
**Total adiponectin, ng/mL** †						
Treatment (*n* = 34)	6070 (3613)	6197 (3954)	127 (−407, 662)	0.59 (0.95)	0.94 (0.99)	0.34 (0.52)
Placebo (*n* = 45)	6424 (4408)	6333 (4385)	−91 (−665, 484)			
**Total osteocalcin, ng/mL**						
Treatment (*n* = 34)	17.5 (6.3)	16.7 (7.7)	−0.8 (−2.3, 0.8)	0.59 (0.15)	0.43 (0.10)	0.58 (0.92)
Placebo (*n* = 45)	20.1 (7.8)	19.4 (8.1)	−0.7 (−2.0, 0.6)			
**Undercarboxylated osteocalcin, ng/mL**						
Treatment (*n* = 34)	11.3 (4.0)	11.0 (4.5)	−0.3 (−1.4, 0.8)	0.70 (0.45)	0.44 (0.10)	0.57 (0.94)
Placebo (*n* = 45)	13.1 (5.0)	12.9 (5.3)	−0.2 (−1.1, 0.6)			

Baseline and 6 month data are presented as mean (SD).

*A multivariate 2-factor repeated-measures regression analysis was used to assess time effects and time by treatment effect interactions on all outcome variables after adjustment for the variables that were different between treatment groups at baseline. Numbers in brackets represent unadjusted *P* values.

† ogarithmically-transformed variables.

‡ *P*<0.1 for the difference between groups at baseline (unpaired *t* test or Wilcoxon, as appropriate).

§ Variables not respecting normality even after transformation are presented as median (25^th^, 75^th^ percentile) and an ANOVA on rank was used to assess treatment effects (adjusted for variables that were different between treatment groups at baseline).

hs-CRP, high sensitive C-reactive protein; IL-6, interleukin-6; NA, not applicable; TNF-α, tumor necrosis factor-alpha.

**Table 5:** Mean baseline and 6 month values and the mean absolute changes in insulin sensitivity, insulin secretion and β-cell function in the subgroup of participants with prediabetes.

			Mean Change	*P* value*		
	Baseline	6 months	(95% CI)	Time	Treatment	Interaction
**HOMA2%S** †						
Treatment (*n* = 13)	58.5 (23.4)	59.0 (25.3)	0.5 (−12.0, 13.1)	0.17	0.04	0.18
Placebo (*n* = 25)	47.6 (22.6)	42.2 (22.5)	−5.5 (−11.0, 0.1)			
**Matsuda index**						
Treatment (*n* = 13)	41.9 (18.7)	44.1 (22.3)	2.1 (−6.2, 10.4)	0.97	0.04	0.29
Placebo (*n* = 25)	32.8 (14.7)	30.8 (13.6)	−2.0 (−6.0, 2.0)			
**Insulinogenic index, µU/mL/mmol/L μµU/mL µU/mL**						
Treatment (*n* = 13)	0.52 (0.21)	0.49 (0.19)	−0.03 (−0.09, 0.03)	0.39	0.84	0.62
Placebo (*n* = 23)	0.52 (0.23)	0.52 (0.23)	−0.01 (−0.07, 0.05)			
**AUC for C-peptide, nmol/L ×120 min**						
Treatment (*n* = 13)	453.7 (167.8)	456.6 (152.8)	2.9 (−30.2, 36.0)	0.65	0.57	0.49
Placebo (*n* = 24)	487.3 (132.1)	473.9 (97.6)	−13.4 (−45.0, 18.2)			
**Disposition index, nmol/L ×120 min** ‡						
Treatment (*n* = 13)	16796 (4760)	17634 (5306)	838 (−1419, 3095)	0.98	0.06	0.17
Placebo (*n* = 24)	14591 (4418)	13730 (5073)	−861 (−2306, 584)			

Baseline and 6 month data are presented as mean (SD).

*A 2-factor repeated-measures regression analysis was used to assess time effects and time by treatment effect interactions on all outcome variables.

† Logarithmically-transformed variables.

‡ Calculated by multiplying Matsuda index by AUC for C-peptide.

AUC, area under the curve; HOMA2%S, Homeostasis Model Assessment 2 index of insulin sensitivity.

### Safety

None of the participants discontinued the study because of side effects associated with treatment. Two participants in the treatment group developed asymptomatic mild hypercalcemia (2.56 and 2.57 mmol/L) at the 2-month visit. Furthermore, five participants in the treatment and two in the placebo group developed hypercalciuria (>10 mmol/d). However, none reported episodes of nephrolithiasis. Other common complaints included: gastrointestinal issues (treatment, *n* = 11; placebo, *n* = 17), fatigue (treatment, *n* = 2; placebo; *n* = 3) and musculoskeletal symptoms (treatment, *n* = 2; placebo, *n* = 2).

## Discussion

In this 6-month RCT of vitamin D and calcium supplementation in which over 90% of the participants reached the target serum 25(OH)D concentration of 75 nmol/L, there was no effect of supplementation on any measure of insulin sensitivity, insulin secretion or β-cell function in multi-ethnic vitamin D-deficient individuals at risk of type 2 diabetes (with prediabetes or an AUSDRISK score ≥15). Moreover, supplementation did not improve cardiovascular disease risk factors such as blood pressure and lipid profile, and had no effect on any inflammatory marker, adiponectin or osteocalcin concentrations. However, in a post hoc analysis restricted to participants with prediabetes (48% of the participants), there was a significant beneficial effect of vitamin D and calcium supplementation on insulin sensitivity, but not on insulin secretion or β-cell function. The results of this pilot study suggest that combined supplementation with calcium and vitamin D in multi-ethnic vitamin D-deficient men and women at risk of type 2 diabetes (based on a diabetes risk questionnaire) may not be an effective strategy to delay or prevent type 2 diabetes. However, in those with prediabetes, there could be a beneficial treatment effect on insulin sensitivity, but these results need to be interpreted with caution given the post hoc nature of the analysis.

The findings from numerous prospective observational studies, including our previous research in over 5,000 Australian adults followed for 5 years, have consistently shown that low vitamin D status is an independent risk factor for type 2 diabetes [3], [4]. However, prospective observational studies looking at the association between insufficient calcium intake and type 2 diabetes risk have shown mixed results [4], [30]–[33]. To our knowledge, only one RCT has evaluated the effects of combined calcium and vitamin D supplementation on insulin sensitivity, insulin secretion and β-cell function in people with glucose intolerance or early diabetes [10], but three others have been conducted in people with type 2 diabetes [6], [9], [18]. In a 2×2 factorial trial of vitamin D_3_ (2,000 IU/d) and calcium (800 mg/d) supplementation for 4 months in 92 mostly Caucasian individuals with prediabetes or early diabetes, Mitri *et al.* found no change in insulin sensitivity in all the treatment groups using the intravenous glucose tolerance test [10]. However, a significant although small improvement in insulin secretion and β-cell function was demonstrated, and there was a trend toward an attenuation of the rise in HbA_1c_ in the vitamin D_3_ supplementation groups, with or without calcium. Supplementation with calcium alone did not change insulin secretion or sensitivity. On the other hand, Nikooyeh *et al.* reported an improvement in insulin resistance (HOMA-IR) in Iranian men and women with type 2 diabetes who consumed a calcium- and vitamin D_3_-fortified yogurt (CDY) containing 1,000 IU of vitamin D_3_ plus 500 mg of calcium daily or a vitamin D_3_-fortified yogurt (DY) containing 1,000 IU of vitamin D_3_ and 300 mg of calcium daily as opposed to a plain yogurt (PY) containing 300 mg of calcium daily for 3 months [6]. Similarly, Shab-Bidar *et al.* found an increase in insulin sensitivity (Quicki) in Iranians with type 2 diabetes who consumed a DY containing 1000 IU of vitamin D_3_ daily versus a PY containing 325 mg of calcium daily for 3 months [9]. In contrast, no change in HOMA-IR was observed in vitamin D-deficient Koreans with type 2 diabetes receiving 2,000 IU of vitamin D_3_ and 200 mg of calcium daily for 6 months [18]. Both the studies by Mitri and Nikooyeh suggest that benefits on glycemic outcomes come from vitamin D and that adding calcium does not provide further improvement. However, of the RCTs of vitamin D supplementation alone that evaluated surrogate markers of type 2 diabetes risk, only three found an increase in insulin sensitivity [5], [7], [8] while the remainder did not find an effect on either insulin sensitivity or secretion [12]–[17], [19]–[23], [34].

The discordant results reported on the effects of vitamin D and/or calcium supplementation on insulin sensitivity and secretion do not seem to be fully explained by factors such as baseline vitamin D status, duration of treatment or vitamin D dose. Indeed, most studies recruited participants with baseline serum 25(OH)D concentrations ranging from 30 to 40 nmol/L. Moreover, positive results on glucose homeostasis have been reported after only 6 weeks of treatment [5] while no effect has been observed after 12 months [13], [15], [17]. In addition, although vitamin D doses varied from 400 IU daily to 88,865 IU per week, several studies, including the current trial, reached a target serum 25(OH)D concentration of >75 nmol/L [8], [15], [34]. A factor that could explain the heterogeneous results obtained from RCTs of vitamin D with or without calcium supplementation is the choice of the population studied in terms of diabetes risk. Our study suggests that those with prediabetes may improve insulin sensitivity with calcium and vitamin D supplementation. However, other studies failed to demonstrate an effect of vitamin D supplementation in this population [11], [15], [22]. People with normal glucose tolerance or at lower risk of type 2 diabetes are unlikely to benefit from treatment [12], [13], [16], [23], [34]. However, it remains unclear whether people with type 2 diabetes may improve glucose homeostasis with calcium and/or vitamin D supplementation [6], [9]–[11], [17]–[21], [35]. Another variable that may explain the discrepant results observed between studies is the presence of polymorphisms in the vitamin D receptor (VDR) gene, and in genes affecting vitamin D and glucose metabolism. Indeed, it was recently demonstrated that individuals presenting certain VDR polymorphisms may be low responders to vitamin D supplementation in terms of improvement in serum 25(OH)D concentrations, insulin sensitivity and inflammation [36], [37]. Although we do not have data to support this, it is possible that common genetic variants affecting glucose homeostasis (e.g. IRS-1) and vitamin D status (e.g. those involved in skin production of vitamin D, in vitamin D-binding protein concentrations and vitamin D catabolism) may also alter treatment response [38]. Finally, baseline calcium intake may also affect response to treatment. Results from Mitri *et al.* [10] and from our study in which total calcium intake was between 1,800 and 2,000 mg daily suggest that increasing total calcium intake above the recommended daily allowance may not improve insulin sensitivity.

We found no beneficial effect of combined calcium and vitamin D supplementation for 6 months on anthropometry, blood pressure, lipid measures, inflammation or metabolic markers. Our results are in line with the majority of previous RCTs that have examined the effect of vitamin D and/or calcium supplementation on cardiovascular disease risk factors [5], [7], [12], [13], [34]. Among the few studies that have shown significant reductions in anthropometric measures, serum triglycerides, inflammatory markers or adiponectin concentrations with supplementation [6], [9], [39], [40], most studied calcium and vitamin D integrated into a yogurt drink [6], [9], suggesting that these benefits may come from dairy. A recent meta-analysis revealed that dairy product consumption reduced body weight and fat [41]. Finally, despite animal and human data suggesting that total and undercarboxylated osteocalcin can regulate glucose homeostasis [42], [43], we found no evidence that vitamin D and calcium supplementation alter this pathogenesis pathway.

Our study has several important strengths. First, this is one of the few RCTs of combined calcium and vitamin D supplementation. Furthermore, we used an escalation regimen of vitamin D that achieved the target serum 25(OH)D concentration of 75 nmol/L in 91% of our participants. It is worth noting that most participants required large doses of vitamin D (4,000 IU per day) to reach this serum 25(OH)D target. It is indeed well known that obese individuals need larger doses of vitamin D, which is possibly due to the sequestration of vitamin D in adipose tissue [44]. Alternatively, the amount of vitamin D contained in the supplements may have been less than what was claimed by the manufacturer but we did not assess this possibility. Finally, we evaluated several cardiometabolic risk factors and investigated potential mechanisms by which vitamin D and calcium could improve insulin secretion and sensitivity.

However, there are several limitations. First, we did not reach our target sample size. We calculated that with 80 participants, our power to detect a difference of 0.86 in delta HOMA-IR between the treatment and placebo groups was 60%. It is thus possible that our study did not have the power to find an effect of treatment. With the small change in HOMA%S that we observed between groups, a sample size of 2,012 participants would be required to find an effect of treatment. Noteworthy, if only participants with prediabetes were recruited, the sample size would be reduced to 285 participants given the larger difference in HOMA%S observed after 6 months in this high-risk group. Future studies should thus aim at recruiting high-risk individuals with prediabetes and plan a longer follow-up. Second, because we did not reach our target sample size, successful randomization was not achieved and unbalanced distributions and proportions of some factors were found between the treatment groups. Although we adjusted our analyses for the variables that were different between treatment groups at baseline, we cannot exclude that residual confounding remains from measured and unmeasured factors, which could invalidate our null results. Third, 16% of the participants dropped out of the study. Although comparison between those who dropped out and those who completed the trial did not reveal differences in baseline characteristics except for age, our selected population may not be representative of the general population. Fourth, none of the participants had a baseline serum 25(OH)D concentration <20 nmol/L and only 10% had a value <30 nmol/L and thus we cannot exclude that vitamin D supplementation would be beneficial in a population with severe vitamin D deficiency. Fifth, our participants received a high total calcium intake from diet and supplements for 6 months. Therefore, we cannot extrapolate our results to a population with low calcium intake. Sixth, we did not assess dietary vitamin A intake, which could potentially interact with vitamin D to alter its efficacy [45]. Moreover, we did not use the gold standard method to evaluate insulin sensitivity, the euglycemic-hyperinsulinemic clamp. Finally, although our study was one of the longest to date, it remains possible that the benefits of calcium and vitamin D supplementation may appear beyond 6 months.

## Conclusions

In conclusion, this study has shown that supplementation with calcium and vitamin D for 6 months may not improve insulin sensitivity, insulin secretion, β-cell function or cardiovascular risk factors in multi-ethnic vitamin D-deficient individuals at risk of type 2 diabetes based on a diabetes risk questionnaire. However, there may be a benefit of treatment on insulin sensitivity in participants with prediabetes. Longer-term RCTs of vitamin D and calcium supplementation focusing on individuals with prediabetes and examining surrogate markers of type 2 diabetes using gold-standard measures as well as measuring the incidence of type 2 diabetes are required to better ascertain whether this could be a safe and effective strategy to prevent type 2 diabetes. Genetic variants also need to be assessed to determine whether subgroups of the population may benefit from this intervention.

## Supporting Information

Checklist S1 **Consort checklist.**
(DOCX)

Protocol S1 **Trial protocol.**
(DOCX)
