# Supplementary material for: Effects of Combined Calcium and Vitamin D Supplementation on Insulin Secretion, Insulin Sensitivity and β-Cell Function in Multi-Ethnic Vitamin D-Deficient Adults at Risk for Type 2 Diabetes: A Pilot Randomized, Placebo-Controlled Trial
Source: PLoS One. 2014 Oct 9;9(10):e109607. doi: 10.1371/journal.pone.0109607 (PMC4192133; doi:10.1371/journal.pone.0109607)
Supplement: Protocol S1 — Trial protocol. (DOCX) [file pone.0109607.s002.docx]

**RESEARCH PROTOCOL**

**Impact of vitamin D and calcium supplementation on the metabolic profile of glucose intolerant and vitamin D deficient obese subjects: a double blind, randomised and controlled trial**

Claudia Gagnon, MD, FRCPC

Peter R. Ebeling, MD, MBBS, FRACP

Leo Rando, MBBS, FRACP

28 February 2008

**1. Introduction**

**1.1 Obesity and Diabetes: Magnitude of the problem**

The results of the last national survey, the Australian Diabetes, obesity and lifestyle study (AusDiab), highlighted the rapid and dramatic rise in the prevalence of diabetes and other categories of abnormal glucose tolerance in Australia [1]. Indeed, almost one in four adult Australians had abnormal glucose tolerance in 2000, one of the highest prevalences reported in a developed nation. This epidemic of diabetes is explained, for the most part, by the coincident doubling in the prevalence of obesity over the last 20 years [2].

Accumulating evidence also suggests that vitamin D and calcium play a physiological role in the modulation of systemic inflammation [3-5], renin-angiotensin system [6], pancreatic β-cell function [7-9] and insulin action [10-14]. It could then be expected that a deregulation of these systems leads to type 2 diabetes, hypertension, metabolic syndrome, and cardiovascular disease (CVD).

**1.2 Observational Studies that Support the link between Vitamin D and Calcium Deficiency and type 2 Diabetes, Metabolic Syndrome and CVD**

Seasonal variation in the incidence of type 2 diabetes [15] and deterioration of glycemic control reported at the end of the winter [16], when vitamin D stores reach their nadir, first pointed toward a possible link between vitamin D deficiency and impaired glucose metabolism. Studies reporting higher prevalence rates of vitamin D deficiency in type 2 diabetics compared to normal controls further supported this hypothesis [5, 17]. It is worth noting that this difference persisted even after adjustment for body-mass index (BMI), which had been shown to independently predict low serum levels of 25-hydroxyvitamin D (25OHD), a reliable marker of vitamin D status [18, 19].

Most experts now consider that the optimal cut-off for 25OHD should be 75 nmol/L [20, 21]. Two recent large cohort studies reinforced the choice of this cut-off for maintenance of normal glucose homeostasis [22, 23]. The first study showed a 60% relative risk reduction (RRR) between the highest and lowest quartiles of 25OHD, which had a mean 25OHD level of 70.9 and 22.4 nmol/L, respectively. The second study showed a 75% RRR between the highest and lowest quartiles of 25OHD (25OHD levels of >80 versus <44 nmol/L, respectively). Recently, an inverse nonlinear correlation, stronger with higher BMIs, was described between 25OHD and HbA_1_C, reaching a plateau at a level of 65 nmol/L [24]. In addition, high serum 25OHD levels have been associated with decreased CVD prevalence among type 2 diabetics, probably mediated by the anti-inflammatory properties of vitamin D [5]. Finally, vitamin D deficiency was the only predictive factor for metabolic syndrome in a population study of severely obese subjects (Odds ratio: 3.1, 95% CI 1.15-8.4, P=0.022) [25].

The protective effect of vitamin D and calcium supplementation on diabetes risk was suggested by the association of high dairy intake with decreased risk of insulin resistance syndrome, mainly in overweight or obese subjects [26]. In the Nurses’ Health Study, a combined daily intake of >1200 mg of calcium and >800 IU of vitamin D conferred the lowest risk of type 2 diabetes (RR: 0.67, 95% CI 0.49-0.9), and was more effective than either taken alone [27]. This effect remained when adjusting for co-variates such as age, BMI and family history.

**1.3 Physiological Roles of vitamin D and Calcium in the Regulation of Glucose homeostasis, Blood Pressure and Inflammation**

***Vitamin D, Calcium and Insulin Secretion***

The discovery of vitamin D receptor and calcium-binding protein expression in pancreatic β cells in the late 1970’s [28, 29], and also more recently, of 1α-25-hydroxylase activity, the enzyme that converts 25OHD into its active metabolite (calcitriol) [30], suggests a role of vitamin D in insulin secretion. Calcium has long been known to play a crucial role in hormone release [31]. Indeed, the first phase of insulin secretion is dependent mainly on β cell intracellular calcium stores whereas the second phase relies both on intra and extracellular calcium stores [32]. Earlier studies in rats showed that vitamin D deficiency impairs insulin secretion, which could be improved by calcitriol supplementation [33-35]. Of note, these studies were performed in vitamin D deficient and hypocalcemic rats. Well-known consequences of vitamin D deficiency are hypocalcemia and secondary hyperparathyroidism. Since these abnormalities could be reversed with adequate vitamin D supplementation, whether the benefits of vitamin D supplementation on insulin secretion could be attributed to vitamin D, independently of its role in calcium and parathormone (PTH) normalisation remains unclear. Indeed, calcium supplementation alone has been shown to increase insulin secretion in vitamin D deplete rats [36, 37]. However, it is reasonable to speculate that the effects of vitamin D and calcium on β cell are additive. Besides its role in maintaining adequate extracellular calcium concentrations, calcitriol has been shown to increase transcription of preproinsulin and calcium-binding proteins (which maintain optimal intracellular calcium reserves for first phase insulin secretion) [38].

***Vitamin D, Calcium and Insulin Sensitivity***

The precise mechanisms through which VD and calcium improve insulin sensitivity are unclear. Calcium has been shown to increase the binding affinity of insulin to its receptor [13] and to promote insulin-mediated glucose transport in adipocytes [14]. The role of VD on insulin sensitivity may rely, at least in part, on a direct stimulatory effect on insulin receptor expression [10] and on modulation of inflammation [3, 5]. Macrophages that reside in the adipose tissue of individuals with type 2 diabetes produce pro-inflammatory cytokines that have been incriminated in the pathophysiology of type 2 diabetes. The expression of VDR and 1α-25-hydroxylase in a wide range of inflammatory cells including monocytes and macrophages results in increased local production of calcitriol inside these cells which then regulates cytokine production by the activated lymphocytes [39]. An *in vitro* study evaluating the effect of VD on isolated monocytes from patients with type 2 diabetes demonstrated that these inflammatory cells decreased their production of pro-inflammatory cytokines such as IL-1, IL-6, IL-8 and TNF-α when exposed to calcitriol [3]. These findings suggest that VD may directly improve insulin sensitivity through modulation of inflammation.

***Osteocalcin and adiponectin as possible novel indirect mediators of effects of vitamin D on insulin sensitivity***

Type 2 diabetes is characterised by the combination of impaired pancreatic β-cell secretion of insulin, and resistance to insulin action in the body. Circulating adiponectin secreted by adipocytes correlates strongly with insulin sensitivity, and negatively with visceral obesity. Furthermore, circulating adiponectin is reduced in patients with type 2 diabetes [40]. Osteocalcin is a novel regulatory mechanism linking bone formation with glucose metabolism via regulation of both β-cell insulin secretion and adiponectin [41]. Mice lacking an osteoblast-expressed receptor-like protein tyrosine phosphatase (*Esp* -/-) displayed increased β-cell proliferation and insulin secretion, and were protected from diabetes. This phenotype was corrected by deletion of a single allele of osteocalcin. In accordance with these findings, osteocalcin knockout mice (*Ocn* -/-) were glucose intolerant and overweight, implicating this osteoblast-secreted circulating molecule as a novel regulator of glucose homeostasis. *Ocn* -/- mice exhibited reduced β-cell mass and pancreatic insulin content, and reduced adiponectin expression and low serum adiponectin levels, similar to humans with type 2 diabetes mellitus. These findings were reversed by treatment with recombinant osteocalcin or co-culture with osteocalcin-expressing fibroblasts. Finally, *Esp* -/- mice had comparable serum osteocalcin as wild-type controls [41], but a lower proportion of gamma-carboxylated osteocalcin, as reflected by reduced binding of serum osteocalcin *in vitro* to hydroxyapatite. Osteocalcin lacking gamma-carboxylation was the metabolically active form, which induced expression of adiponectin in adipocytes. These findings represent a new paradigm of regulation of energy metabolism by the skeleton, with non-gamma-carboxylated osteocalcin acting as a new hormone and systemic stimulator of insulin and adiponectin secretion [42]. VD may also have a role in gamma-carboxylation of osteocalcin [43].

***Vitamin D and Regulation of the Renin-Angiotensin System***

Finally, calcitriol appears to be a strong down-regulator of the renin-angiotensin system through direct suppression of the renin gene promoter activity [6]. Indeed, renin expression and angiotensin II concentration are increased in VDR-null mice, leading to hypertension.

**1.4 Effects of vitamin D and Calcium Supplementation in Humans**

Inconsistent results have been reported from the few studies that evaluated the effect of vitamin D supplementation on glucose homeostasis [44-50]. In summary, no clear effect of vitamin D supplementation on insulin secretion or insulin sensitivity was found in lean, normal glucose tolerant and vitamin D replete subjects [46]. Most studies of glucose intolerant and vitamin D sufficient individuals have not shown a benefit from treatment with vitamin D [48, 49]. However, one study showed that vitamin D supplementation in severely vitamin D deficient Asians at high risk for type 2 diabetes (95% of the population had 25OHD levels < 24 nmol/L) increased insulin and C-peptide levels post glucose load compared to vitamin D deficient individuals at low risk for the disease [47]. A significant increase in first phase insulin secretion and non significant increase in second phase insulin secretion and insulin resistance was also noted after vitamin D supplementation in a small sample of vitamin D deficient type 2 diabetic females [50]. Only one study in humans evaluated the combined effect of vitamin D and calcium supplementation on glucose homeostasis [45]. Of note, this study was designed for primary skeletal outcomes. However, treatment with 700 IU of vitamin D_3_ and 500 mg of elemental calcium for 3 years in overweight and vitamin D sufficient people with impaired fasting glucose prevented the increase in fasting plasma glucose and insulin resistance observed in the placebo group.

**2. Rationale of Project**

Taken together, evidences from *in vitro*, animal and observational human studies suggest that vitamin D and calcium supplementation improve the metabolic profile of vitamin D deficient individuals. It is also reasonable to expect from the physiological roles calcium and vitamin D seem to play on insulin secretion and sensitivity that their effects are additive. However, calcium and vitamin D supplementation trials have yielded contradicting results. Many factors could explain this discrepancy. First, the baseline vitamin D and glucose tolerance status of the selected populations is very heterogeneous between studies. In addition, the main shortcoming of these papers is the lack of ascertainment of adequate vitamin D supplementation. Targeting optimal 25OHD levels is particularly important, since based on observational studies, an improvement in glucose homeostasis is expected at 25OHD levels over 65-80 nmol/L [22-24]. To conclude from these studies, we expect that the group to benefit most from the intervention would be those with vitamin D deficiency at high risk for type 2 diabetes. Those in the early stages of the disease, when irreversible β cell failure has not yet occurred, would seem appropriate to target due to the beneficial effects seen on insulin secretion.

To our knowledge, the study we propose is the first specifically designed to assess the combined effect of adequate calcium and vitamin D supplementation on glucose homeostasis and metabolic profile on vitamin D deficient subjects at high risk of developing type 2 diabetes. If a beneficial effect of treatment is demonstrated, it could have major implications on the prevention and treatment of this escalating disease.

**3. Aims/Hypothesis**

**3.1 Primary Aim**

The primary aim of this study is to determine if adequate vitamin D supplementation (2000-4000 IU of vitamin D_3_ daily, assessed by a serum 25OHD level of ≥75 nmol/L) and calcium supplementation (1200 mg daily) in vitamin D deficient individuals at high risk of type 2 diabetes improve:

- Insulin sensitivity (homeostasis model assessment of insulin resistance – HOMA-IR);
- Glucose-stimulated insulin secretion (insulinogenic index and deconvolution analysis of C-peptide levels after a glucose load);
- Beta-cell function (disposition index).

**Primary Hypothesis:** Combined treatment with VD and calcium will significantly improve the insulin sensitivity, glucose-stimulated insulin secretion and β-cell function of VD deficient men and women at high risk of type 2 diabetes compared with placebo.

**3.2 Secondary Aims**

- Secondary objectives are to assess, in this population of vitamin D deficient individuals at high risk of type 2 diabetes, the effect of treatment on:
  1. Inflammatory markers;
  2. Adiponectin and osteocalcin;
  3. Insulin resistance using the revised Quicki index;
  4. Blood pressure;
  5. Lipid profile;
  6. Fasting free-fatty acid (FFA) levels;
  7. Body weight.
- We also seek to determine whether vitamin D requirements (to achieve a level of 25OHD ≥75 nmol/L) are influenced by fat mass (dual X-ray absorptiometry - DXA) in this population of overweight individuals.

**5. Project Design**

**5.1 Study Population/Recruitment strategies**

Eighty (80) overweight or obese, vitamin D deficient and glucose intolerant subjects with various ethnic backgrounds will be enrolled in this study. Most participants will be recruited from local General Practices. Recruitment via the GP clinics will be done in two phases.

- First phase: The Diabetes Risk Score questionnaire, a simple tool developed from large epidemiological studies, will be used to maximize the yield of finding glucose intolerant subjects. In one recently published study, about a third of the individuals with a screening score of 12 or more turned out to be glucose intolerants [51]. This cut-off will therefore be used to identify patients for OGTT testing. This simple self-administered questionnaire as well as an invitation to participate in the study will be given by the GPs secretary when the patient registers for his appointment. If the patient agrees to be contacted (by giving his contact details), he will give this letter back to the secretary that will send it to the research team. The research team of Western hospital will then contact the patient to make an appointment (to explain the protocol, sign the consent form and proceed with the screening OGTT). Advertisements will also be put on the walls of the clinics waiting room.
- Phase 2 (this strategy will be only applied if the phase 1 recruitment strategies have failed. A separate consent form will be used for this recruitment strategy): After presenting the project to GP groups and obtained their agreement, a nurse or the study investigators will screen patients in the clinic waiting room. While patients will be waiting for their appointment with their GP, the researchers (Claudia Gagnon) or the research assistant will ask if they would like to participate in the study. If they agree, they will be transferred in a private room to read the consent form and sign it. They will then have their weight, height and waist circumference. These measurements will be used to answer the Diabetes Risk Score questionnaire. If the score of the patient is 12 or more, he (she) will be given an appointment for an OGTT. The researchers and the GPs will receive the results of this test and contact the patient if he (she) is eligible to participate in this study. Of note, by signing this version of the consent form (only for recruitment of participants from GP clinics – Phase 2), the patient accepts that the researchers and the GPs receive a copy of the OGTT results, access their medical record and contact them if they are eligible to participate in the study. The subsequent visits will be held at Western Hospital.

Participants will also be recruited from outpatient clinics in Western Health (Sunshine, Footscray and Williamstown campuses) and Royal Melbourne Hospital (RMH). More specifically, patients who are on the waiting list for obesity clinic at RMH and Western hospital will be invited by mail to participate in this study. In addition, patients who had gestational diabetes and attended the gestational diabetes clinic in the last 5 years will also receive an invitation. Finally, announcements will be made in local newspapers, radio and also displayed in the above mentioned hospitals, in strategic locations planned for this purpose.

**5.2 Randomisation and Intervention**

The proposed study is a prospective, double blind, randomised and placebo controlled 6-month clinical trial. Subjects will be randomly assigned to one of two groups, with stratification according to sex, age (< or >50 years of age) and BMI (< or >30). The treatment group will receive vitamin D_3_ (cholecalciferol) 2000 IU plus calcium carbonate (1200 mg elemental Ca daily) and the control group will take placebo pills. Permuted blocks of 4 treatment allocations will be used to ensure that after each 4^th^ allocation there are equal numbers in each treatment arm. After 8 weeks of treatment, if subjects in the treatment group have 25OHD levels less than 75 nmol/L, the dose of cholecalciferol will be increased to 4000 IU daily to reach optimal levels. On the other hand, subjects in the placebo group and those in the treatment group that reached optimal levels of 25OHD will be given extra placebo pills. To ensure blinding is respected, a person external to the study will look at the 8-week 25OHD results and contact patients to add an extra cholecalciferol or placebo pill to their regime, as appropriate.

The dosage of cholecalciferol has been estimated from the study of Barger-Lux and al., a rigorous trial performed in healthy overweight men [52]. They showed that each mcg (40 IU) of vitamin D_3_ increase the 25OHD concentration by 0.70 nmol/L. Based on the mean 25OHD level reported in populations of obese vitamin D deficient subjects (30 nmol/L) [25, 50, 53] and the expected increment described previously, it can be estimated that 2400 IU daily of vitamin D_3_ would be needed to increase the mean 25OHD level of our population to over 75 nmol/L. Of note, the dose-response relationship was not affected by body weight or fat mass. However, other data suggest that vitamin D requirements might be higher in obese compared to normal weight subjects [54]. Therefore, given that participants in our study will be obese, if adequate 25OHD levels are not reached after 8 weeks, the dose of cholecalciferol will be increased to 4000 IU daily. This dose, given daily for 3 months to 28 healthy adults, increased mean 25OHD levels from 37.9 ± 13.4 nmol/L to 96.4 ± 14.6 nmol/L [55]. None of the subjects had 25OHD levels <75 nmol/L at the end of the study.

**5.3 Inclusion Criteria**

1. Age ≥ 18 years;
2. Any ethnicity;
3. Vitamin D deficiency documented by a serum 25OHD concentration ≤ 50 nmol/L;
4. Overweight or obesity (BMI ≥ 25 kg/m^2^ and ≤ 40 kg/m^2^);
5. Glucose intolerance confirmed with a 75g OGTT in the last 3 months: fasting plasma glucose < 7.0 mmol/L and/or 2h post glucose load between 7.8 and 11.0 mmol/L (WHO criteria 1999);
6. Ability to give an informed consent.

**5.4 Exclusion Criteria**

1. Pregnancy or intention to become pregnant in the next 6 months;
2. Breast-feeding;
3. Renal insufficiency (creatinine clearance < 60 ml/minute);
4. Cirrhosis;
5. Malabsorption*;
6. Known history of hypercalcemia, hypercalciuria or nephrolithiasis;
7. Previous fractures;
8. Known active inflammatory disease;
9. Medications known to affect vitamin D, calcium or bone metabolism, such as vitamin D and calcium supplements, bisphosphonates, glucocorticoids, thiazide diuretics, anticonvulsivants and lithium over the last 3 months;
10. Pharmacological treatment for obesity or medications known to alter glucose metabolism over the last 3 months;
11. Bariatric surgery planned in the next six months or patients who had undergone bariatric surgery in the past;
12. More than 5% change in weight in the last 3 months;
13. Current smoking.

* Every participant will be screened for celiac disease (anti-tissue transglutaminase antibodies). Those with positive results will be excluded from the study and referred to their family physician for further investigation and treatment.

**5.5 Procedures**

Pre-randomisation measurements

To ensure the inclusion and exclusion criteria are met, a 24h urine collection for calcium and blood tests for 25OHD, calcium, albumin and renal function must have been done in the last month. A 75g 2h OGTT result no more than 3 months old must also be available.

Measurements at baseline and at the end of the study

*Anthropometrical and blood pressure assessments**

- Height (to the nearest 0.5 cm), using a wall-mounted stadiometer;
- Body weight (to the nearest 0.1 kg), measured without shoes and in light clothing, using an electronic calibrated scale;
- Waist circumference (to the nearest 0.5 cm), using a tape placed at the midpoint between the lower rib and the upper iliac crest;
- Blood pressure, taken 3 times in the sitting position with an adequate pressure cuff, after a 5-minute rest. The mean of the 3 results will be used for statistical purposes.

*These parameters will also be measured at 2 months

*Body composition*

Fat mass will be measured using dual-energy X-ray absorptiometry (Hologic, Discovery W, Bedford, MA, USA) only at the beginning of the study to correlate fat mass with the dose of vitamin D_3_ needed to reach optimal 25OHD levels.

*Biochemical assessments*

- Fasting blood will be drawn for 25OHD, total calcium, albumin, phosphate, PTH, alkaline phosphatase, osteocalcin, complete lipid profile, HbA_1_C, FFAs, adiponectin, high-sensitive C-reactive protein (hs-CRP) and fibrinogen;
- 2h OGTT after 75g of glucose: glucose, insulin and C-peptide levels will be measured at times -30, -20, -10, 0, 10, 20, 30, 60, 90 and 120 minutes;
- 24h urinary calcium and creatinine;
- Extra aliquot for storage for measurement of other bone markers and markers of inflammation (IL-6 and TNF-alpha) and diabetes when the funds will be available.

*Questionnaires*

- Medical history;
- Food questionnaire to estimate vitamin D and calcium intake [56];
- Physical activity questionnaire [57];
- Questionnaire to estimate sunlight exposure.

*Assessment of β-cell insulin secretion, insulin resistance and beta-cell function*

- **Insulin secretion rate** will be estimated by two methods:
  - The insulinogenic index ([Ins_t30_ – Ins_t0_] ÷ [Glucose_t30_ – Glucose_t0_]), which mainly reflects the first phase of insulin secretion;
  - The deconvolution analysis of C-peptide levels after a glucose load. C-peptide is probably a better indicator of insulin secretion than insulin itself since the latter is subject to hepatic clearance. Pre-hepatic ISR will be calculated from deconvolution of peripheral plasma C-peptide levels using a two compartment mathematical model with standard parameters for C-peptide distribution and metabolism, as previously described using the Chronobiological Series Analyses software (iBridge Network, [www.iBridgeNetwork.org](http://www.iBridgeNetwork.org), University of Chicago) [58]. Use of standard parameters for C-peptide clearance and distribution has been shown to result in insulin secretion rates that differ in each subject by only 10-12% from those obtained with individual parameters and there is no systematic over- or underestimation of insulin secretion. Incremental ISR from baseline during the first 30 minutes divided by incremental glucose and absolute total area under the curve of C-peptide by deconvolution analysis will be compared between baseline and 6 months of treatment.
- **Insulin resistance** will also be assessed by two methods:
  - The homeostasis model assessment for insulin resistance (HOMA-IR) is particularly suitable for obese and/or glucose intolerant subjects and has been shown to highly correlate with measures of insulin sensitivity obtained from the gold-standard procedure (euglycemic clamp technique) [59-61]. It is calculated from fasting insulin and glucose levels as follows: [Fasting insulin (mU/L) X fasting glucose (mmol/L)] ÷ 22.5.
  - Insulin resistance will also be estimated with the revised Quicki index: 1/[log (fasting glucose in mg/dl) + log (fasting insulin in μunits/ml) + log (fasting FFA). This index was shown to better correlate with euglycemic clamp than HOMA-IR in impaired glucose tolerant individuals (r=0,802) [62].
- **Beta-cell function** will be estimated by calculating the disposition index (DI): DI = insulin sensitivity (HOMA-IR) X ISR (deconvolution analysis of C-peptides).

*Evaluation of compliance*

Pill count will be performed after two months and at the end of the study to verify compliance to medication. Participants will be asked to report any change in their medication since the last visit.

*Monitoring of side effects and Safety Issues*

Vitamin D replacement therapy is very safe. Cholecalciferol doses up to 10 000 IU daily have not caused hypercalcemia *per se* in clinical trials [63]. Screening laboratory tests will ensure that subjects with hypercalcemia are not included in the study. Furthermore, at two and 6 months, monitoring of serum 25OHD and calcium levels will be done. Participants will also be instructed about the symptoms of hypercalcemia and asked to contact the research team immediately if these symptoms develop during the course of the study.

**5.6 Statistical analyses**

Analyses will be performed using SPSS version 15.0. Skewed data will be log-transformed or examined using non-parametric tests. Differences in delta HOMA-IR between the treatment and the placebo group will be compared by two-sample unpaired *t* test. The change in DI and in ISR (assessed by deconvolution analysis of C-peptide levels and the insulinogenic index) and also the total area under the curve of C-peptide by deconvolution analysis will be compared between groups with unpaired *t* test. Analyses will be performed to assess changes in inflammatory markers, intact osteocalcin, adiponectin, blood pressure, lipid profile, FFA and weight with a two-sample unpaired *t* test. A *P*-value of ≤0.05 will be considered statistically significant. The endpoints will be adjusted for risk factors of type 2 diabetes: age, sex, BMI, family history of type 2 diabetes and physical activity. The following covariates will also be adjusted for: sunlight exposure and VD and calcium intake. Finally, the variables that predict response to treatment will be identified with multiple linear regression analyses. We will perform data analysis on an intention-to-treat principle. We will also perform analysis on patients who achieved >75% compliance with study medication as well as those on active treatment who achieved target 25OHD levels ≥75 nmol/L.

*Sample size calculation*

Sample size calculation is based on the difference in delta HOMA-IR [0.15 ± 0.18 (SD)] observed in a similar population of overweight subjects with pre-diabetes before and after a mean weight loss of 9.8 kg [64]. This change in HOMA-IR resulted in improved beta-cell function and reduction in the area under glucose curve during OGTT. This change in HOMA-IR was therefore considered clinically significant and was chosen for sample size calculation. A sample of 32 participants in each group will have 90% power to detect a difference in delta HOMA-IR of 0.15 with a standard deviation of 0.18 and 2-sided alpha of 0.05. Assuming a drop out rate of 20%, we will study a total of 80 participants.

**5.7 Limitations**

None.

**5.8 Potential sources of bias**

The main factors that could affect insulin sensitivity, and consequently, insulin secretion are physical activity and weight change. Physical activity will be evaluated with questionnaires at the beginning and at the end of the study. This factor will be taken into account in the analyses. However, some data suggest that calcium supplementation could induce weight loss [65, 66]. If significant weight loss is observed between the treatment and placebo group, it will be difficult to discriminate the beneficial effects of calcium and vitamin D on insulin sensitivity and secretion from those induced by weight loss. However, this will be adjusted for in multivariate analysis.

Seasonal variation can influence endogenous 25OHD production. With small number block randomisation, this effect will be minimised by distributing patients equally between treatment groups within a small time frame.

1. Dunstan, D.W., et al., *The rising prevalence of diabetes and impaired glucose tolerance: the Australian Diabetes, Obesity and Lifestyle Study.* Diabetes Care, 2002. **25**(5): p. 829-34.
2. Cameron, A.J., et al., *Overweight and obesity in Australia: the 1999-2000 Australian Diabetes, Obesity and Lifestyle Study (AusDiab).* Med J Aust, 2003. **178**(9): p. 427-32.
3. Giulietti, A., et al., *Monocytes from type 2 diabetic patients have a pro-inflammatory profile. 1,25-Dihydroxyvitamin D(3) works as anti-inflammatory.* Diabetes Res Clin Pract, 2007. **77**(1): p. 47-57.
4. Targher, G., et al., *Serum 25-hydroxyvitamin D3 concentrations and carotid artery intima-media thickness among type 2 diabetic patients.* Clin Endocrinol (Oxf), 2006. **65**(5): p. 593-7.
5. Cigolini, M., et al., *Serum 25-hydroxyvitamin D3 concentrations and prevalence of cardiovascular disease among type 2 diabetic patients.* Diabetes Care, 2006. **29**(3): p. 722-4.
6. Li, Y.C., et al., *1,25-Dihydroxyvitamin D(3) is a negative endocrine regulator of the renin-angiotensin system.* J Clin Invest, 2002. **110**(2): p. 229-38.
7. Billaudel, B., L. Barakat, and A. Faure-Dussert, *Vitamin D3 deficiency and alterations of glucose metabolism in rat endocrine pancreas.* Diabetes Metab, 1998. **24**(4): p. 344-50.
8. Tanaka, Y., et al., *Effect of vitamin D3 on the pancreatic secretion of insulin and somatostatin.* Acta Endocrinol (Copenh), 1984. **105**(4): p. 528-33.
9. Chiu, K.C., et al., *Hypovitaminosis D is associated with insulin resistance and beta cell dysfunction.* Am J Clin Nutr, 2004. **79**(5): p. 820-5.
10. Maestro, B., et al., *Stimulation by 1,25-dihydroxyvitamin D3 of insulin receptor expression and insulin responsiveness for glucose transport in U-937 human promonocytic cells.* Endocr J, 2000. **47**(4): p. 383-91.
11. Ojuka, E.O., *Role of calcium and AMP kinase in the regulation of mitochondrial biogenesis and GLUT4 levels in muscle.* Proc Nutr Soc, 2004. **63**(2): p. 275-8.
12. Wright, D.C., et al., *Ca2+ and AMPK both mediate stimulation of glucose transport by muscle contractions.* Diabetes, 2004. **53**(2): p. 330-5.
13. Williams, P.F., et al., *High affinity insulin binding and insulin receptor-effector coupling: modulation by Ca2+.* Cell Calcium, 1990. **11**(8): p. 547-56.
14. Segal, S., et al., *Postprandial changes in cytosolic free calcium and glucose uptake in adipocytes in obesity and non-insulin-dependent diabetes mellitus.* Horm Res, 1990. **34**(1): p. 39-44.
15. Doro, P., et al., *Seasonality in the incidence of type 2 diabetes: a population-based study.* Diabetes Care, 2006. **29**(1): p. 173.
16. Ishii, H., et al., *Seasonal variation of glycemic control in type 2 diabetic patients.* Diabetes Care, 2001. **24**(8): p. 1503.
17. Scragg, R., et al., *Serum 25-hydroxyvitamin D3 levels decreased in impaired glucose tolerance and diabetes mellitus.* Diabetes Res Clin Pract, 1995. **27**(3): p. 181-8.
18. Snijder, M.B., et al., *Adiposity in relation to vitamin D status and parathyroid hormone levels: a population-based study in older men and women.* J Clin Endocrinol Metab, 2005. **90**(7): p. 4119-23.
19. Bischof, M.G., G. Heinze, and H. Vierhapper, *Vitamin D status and its relation to age and body mass index.* Horm Res, 2006. **66**(5): p. 211-5.
20. Bischoff-Ferrari, H.A., et al., *Estimation of optimal serum concentrations of 25-hydroxyvitamin D for multiple health outcomes.* Am J Clin Nutr, 2006. **84**(1): p. 18-28.
21. Holick, M.F., *Vitamin D deficiency.* N Engl J Med, 2007. **357**(3): p. 266-81.
22. Mattila, C., et al., *Serum 25-hydroxyvitamin D concentration and subsequent risk of type 2 diabetes.* Diabetes Care, 2007. **30**(10): p. 2569-70.
23. Scragg, R., M. Sowers, and C. Bell, *Serum 25-hydroxyvitamin D, diabetes, and ethnicity in the Third National Health and Nutrition Examination Survey.* Diabetes Care, 2004. **27**(12): p. 2813-8.
24. Hypponen, E. and C. Power, *Vitamin D status and glucose homeostasis in the 1958 British birth cohort: the role of obesity.* Diabetes Care, 2006. **29**(10): p. 2244-6.
25. Botella-Carretero, J.I., et al., *Vitamin D deficiency is associated with the metabolic syndrome in morbid obesity.* Clin Nutr, 2007. **26**(5): p. 573-80.
26. Pereira, M.A., et al., *Dairy consumption, obesity, and the insulin resistance syndrome in young adults: the CARDIA Study.* Jama, 2002. **287**(16): p. 2081-9.
27. Pittas, A.G., et al., *Vitamin D and calcium intake in relation to type 2 diabetes in women.* Diabetes Care, 2006. **29**(3): p. 650-6.
28. Christakos, S. and A.W. Norman, *Studies on the mode of action of calciferol. XVIII. Evidence for a specific high affinity binding protein for 1,25 dihydroxyvitamin D3 in chick kidney and pancreas.* Biochem Biophys Res Commun, 1979. **89**(1): p. 56-63.
29. Morrissey, R.L., et al., *Calcium-binding protein: its cellular localization in jejunum, kidney and pancreas.* Proc Soc Exp Biol Med, 1975. **149**(1): p. 56-60.
30. Bland, R., et al., *Expression of 25-hydroxyvitamin D3-1alpha-hydroxylase in pancreatic islets.* J Steroid Biochem Mol Biol, 2004. **89-90**(1-5): p. 121-5.
31. Wollheim, C.B., et al., *The roles of intracellular and extracellular Ca++ in glucose-stimulated biphasic insulin release by rat islets.* J Clin Invest, 1978. **62**(2): p. 451-8.
32. Kikuchi, M., et al., *Biphasic insulin release in rat islets of Langerhans and the role of Intracellular Ca++ stores.* Endocrinology, 1979. **105**(4): p. 1013-9.
33. Cade, C. and A.W. Norman, *Vitamin D3 improves impaired glucose tolerance and insulin secretion in the vitamin D-deficient rat in vivo.* Endocrinology, 1986. **119**(1): p. 84-90.
34. Cade, C. and A.W. Norman, *Rapid normalization/stimulation by 1,25-dihydroxyvitamin D3 of insulin secretion and glucose tolerance in the vitamin D-deficient rat.* Endocrinology, 1987. **120**(4): p. 1490-7.
35. de Souza Santos, R. and L.M. Vianna, *Effect of cholecalciferol supplementation on blood glucose in an experimental model of type 2 diabetes mellitus in spontaneously hypertensive rats and Wistar rats.* Clin Chim Acta, 2005. **358**(1-2): p. 146-50.
36. Ismail, A. and R. Namala, *Impaired glucose tolerance in vitamin D deficiency can be corrected by calcium.* J Nutr Biochem, 2000. **11**(3): p. 170-5.
37. Beaulieu, C., et al., *Calcium is essential in normalizing intolerance to glucose that accompanies vitamin D depletion in vivo.* Diabetes, 1993. **42**(1): p. 35-43.
38. Ozono, K., et al., *1,25-Dihydroxyvitamin D3 enhances the effect of refeeding on steady state preproinsulin messenger ribonucleic acid levels in rats.* Endocrinology, 1990. **126**(4): p. 2041-5.
39. Veldman, C.M., M.T. Cantorna, and H.F. DeLuca, *Expression of 1,25-dihydroxyvitamin D(3) receptor in the immune system.* Arch Biochem Biophys, 2000. **374**(2): p. 334-8.
40. Weyer, C., et al., *Hypoadiponectinemia in obesity and type 2 diabetes: close association with insulin resistance and hyperinsulinemia.* J Clin Endocrinol Metab, 2001. **86**(5): p. 1930-5.
41. Lee,N.K,et al.,*Endocrine regulation of energy metabolism by the skeleton.*Cell,2007.**130**(3)456-69.
42. Rosen,CJ,*Bone remodeling,energy metabolism,and the molecular clock.*Cell Metab,2008.**7**(1)7-10.
43. Deyl, Z. and M. Adam, *Evidence for vitamin D dependent gamma-carboxylation in osteocalcin-related proteins.* Biochem Biophys Res Commun, 1983. **113**: p. 294-300.
44. Orwoll, E., M. Riddle, and M. Prince, *Effects of vitamin D on insulin and glucagon secretion in non-insulin-dependent diabetes mellitus.* Am J Clin Nutr, 1994. **59**(5): p. 1083-7.
45. Pittas, A.G., et al., *The effects of calcium and vitamin D supplementation on blood glucose and markers of inflammation in nondiabetic adults.* Diabetes Care, 2007. **30**(4): p. 980-6.
46. Fliser, D., et al., *No effect of calcitriol on insulin-mediated glucose uptake in healthy subjects.* Eur J Clin Invest, 1997. **27**(7): p. 629-33.
47. Boucher, B.J., et al., *Glucose intolerance and impairment of insulin secretion in relation to vitamin D deficiency in east London Asians.* Diabetologia, 1995. **38**(10): p. 1239-45.
48. Ljunghall, S., et al., *Treatment with one-alpha-hydroxycholecalciferol in middle-aged men with impaired glucose tolerance--a prospective randomized double-blind study.* Acta Med Scand, 1987. **222**(4): p. 361-7.
49. Lind, L., et al., *Long-term treatment with active vitamin D (alphacalcidol) in middle-aged men with impaired glucose tolerance. Effects on insulin secretion and sensitivity, glucose tolerance and blood pressure.* Diabetes Res, 1989. **11**(3): p. 141-7.
50. Borissova, A.M., et al., *The effect of vitamin D3 on insulin secretion and peripheral insulin sensitivity in type 2 diabetic patients.* Int J Clin Pract, 2003. **57**(4): p. 258-61.
51. Laatikainen T.,et al., *Prevention of type 2 diabetes by lifestyle intervention in an Australian primary health care setting: Greater Green Triangle Diabetes Prevention Project.* BMC Public Health, 2007. **7**: 249-55.
52. Heaney, R.P., et al., *Human serum 25-hydroxycholecalciferol response to extended oral dosing with cholecalciferol.* Am J Clin Nutr, 2003. **77**(1): p. 204-10.
53. Liel, Y., et al., *Low circulating vitamin D in obesity.* Calcif Tissue Int, 1988. **43**(4): p. 199-201.
54. Wortsman,J, et al, *Decreased bioavailability of vitamin D in obesity.* Am J Clin Nutr,2000. **72**:690-3.
55. Vieth, R., P.C. Chan, and G.D. MacFarlane, *Efficacy and safety of vitamin D3 intake exceeding the lowest observed adverse effect level.* Am J Clin Nutr, 2001. **73**(2): p. 288-94.
56. Pasco, J.A., et al., *Calcium intakes among Australian women: Geelong Osteoporosis Study.* Aust N Z J Med, 2000. **30**(1): p. 21-7.
57. *National Physical Activity Survey*. 1999, The Australian Institute of Health and Welfare. p. 32-49.
58. Van Cauter, E., et al., *Estimation of insulin secretion rates from C-peptide levels. Comparison of individual and standard kinetic parameters for C-peptide clearance.* Diabetes, 1992. **41**(3): p. 368-77.
59. Matthews, D.R., et al., *Homeostasis model assessment: insulin resistance and beta-cell function from fasting plasma glucose and insulin concentrations in man.* Diabetologia, 1985. **28**(7): p. 412-9.
60. Matsuda, M. and R.A. DeFronzo, *Insulin sensitivity indices obtained from oral glucose tolerance testing: comparison with the euglycemic insulin clamp.* Diabetes Care, 1999. **22**(9): p. 1462-70.
61. Emoto, M., et al., *Homeostasis model assessment as a clinical index of insulin resistance in type 2 diabetic patients treated with sulfonylureas.* Diabetes Care, 1999. **22**(5): p. 818-22.
62. Rabasa-Lhoret, R., et al., *Modified quantitative insulin sensitivity check index is better correlated to hyperinsulinemic glucose clamp than other fasting-based index of insulin sensitivity in different insulin-resistant states.* J Clin Endocrinol Metab, 2003. **88**(10): p. 4917-23.
63. Byrne, P.M., R. Freaney, and M.J. McKenna, *Vitamin D supplementation in the elderly: review of safety and effectiveness of different regimes.* Calcif Tissue Int, 1995. **56**(6): p. 518-20.
64. Couture, C., et al., *Weight loss is associated with improvement of beta cell function in subjects with impaired glucose tolerance.* Obesity, 2007. **15**(Suppl.): A186.
65. Zemel, M.B., et al., *Calcium and dairy acceleration of weight and fat loss during energy restriction in obese adults.* Obes Res, 2004. **12**(4): p. 582-90.
66. Major, G.C., et al., *Supplementation with calcium + vitamin D enhances the beneficial effect of weight loss on plasma lipid and lipoprotein concentrations.* Am J Clin Nutr, 2007. **85**(1): p. 54-9.
